# A Novel Therapy to Attenuate Acute Kidney Injury and Ischemic Allograft Damage after Allogenic Kidney Transplantation in Mice

**DOI:** 10.1371/journal.pone.0115709

**Published:** 2015-01-24

**Authors:** Faikah Gueler, Nelli Shushakova, Michael Mengel, Katja Hueper, Rongjun Chen, Xiaokun Liu, Joon-Keun Park, Hermann Haller, Gert Wensvoort, Song Rong

**Affiliations:** 1 Department of Nephrology, Hannover Medical School, Hannover, Germany; 2 Phenos GmbH, Hannover, Germany; 3 Department of Laboratory Medicine & Pathology, University of Alberta, Edmonton, Canada; 4 Diagnostic and Investigative Radiology, Hannover Medical School, Hannover, Germany; 5 The kidney disease centre of the First Affiliated Hospital, Zhejiang University, Hangzhou, China; 6 Department of Gastrointestinal Surgery, First Affiliated Hospital, Zhejiang University, Hangzhou, China; 7 Exponential Biotherapies Inc., The Hague, The Netherlands; 8 The Transplantation Center of the affiliated hospital, Zunyi Medical College, Zunyi, China

## Abstract

Ischemia followed by reperfusion contributes to the initial damage to allografts after kidney transplantation (ktx). In this study we tested the hypothesis that a tetrapeptide EA-230 (AQGV), might improve survival and attenuate loss of kidney function in a mouse model of renal ischemia/reperfusion injury (IRI) and ischemia-induced delayed graft function after allogenic kidney transplantation. IRI was induced in male C57Bl/6N mice by transient bilateral renal pedicle clamping for 35 min. Treatment with EA-230 (20–50mg/kg twice daily i.p. for four consecutive days) was initiated 24 hours after IRI when acute kidney injury (AKI) was already established. The treatment resulted in markedly improved survival in a dose dependent manner. Acute tubular injury two days after IRI was diminished and tubular epithelial cell proliferation was significantly enhanced by EA-230 treatment. Furthermore, CTGF up-regulation, a marker of post-ischemic fibrosis, at four weeks after IRI was significantly less in EA-230 treated renal tissue. To learn more about these effects, we measured renal blood flow (RBF) and glomerular filtration rate (GFR) at 28 hours after IRI. EA-230 improved both GFR and RBF significantly. Next, EA-230 treatment was tested in a model of ischemia-induced delayed graft function after allogenic kidney transplantation. The recipients were treated with EA-230 (50 mg/kg) twice daily i.p. which improved renal function and allograft survival by attenuating ischemic allograft damage. In conclusion, EA-230 is a novel and promising therapeutic agent for treating acute kidney injury and preventing IRI-induced post-transplant ischemic allograft injury. Its beneficial effect is associated with improved renal perfusion after IRI and enhanced regeneration of tubular epithelial cells.

## Introduction

Ischemia reperfusion injury (IRI) causes acute kidney injury (AKI) with tubular and endothelial damage and leads to an early loss of peritubular capillaries (PTC) [1], decreased renal perfusion, and inflammation and fibrosis of the kidney [2]. In cadaveric kidney transplantation, ischemic allograft damage is correlated with impaired microcirculation in the peritubular capillaries [3] resulting in delayed graft function (DGF) [4]. DGF is a form of acute kidney injury (AKI) causing post-transplantation oliguria and increased allograft immunogenicity. It is associated with an increased risk of acute rejection episodes, and decreased long-term survival [5]. In addition, AKI contributes to increased morbidity and mortality following the transplantation of organs other than the kidney. In lung transplantation [6,7], and non-myeloablative hematopoietic cell transplantation AKI is seen in more than 50% of patients and it increases the risk of mortality [8].

Impaired renal blood flow is a critical event after IRI. It initiates inflammation, capillary leakage, and microcirculation disturbances, and contributes to fibrosis. Functional MRI techniques have allowed us to correlate early microcirculation impairment after IRI and the extent of renal damage and kidney volume loss over time [9,10]. Standard immunosuppressive therapies in organ transplantation do not address the early ischemic injury or prevent AKI. Therefore, new therapeutic options to address microcirculation impairment, inflammation and early graft injury are needed.

Several synthetic oligopeptides originally derived from beta-human chorionic gonadotropin (beta-hCG) lysates, have been shown to have anti-inflammatory therapeutic effects in mouse models of LPS and CLP (cecum ligation and puncture) induced sepsis [11,12]. They increased survival and reduced systemic release of pro-inflammatory cytokines such as TNF-alpha, IL-1 and IL-6 [12]. In the IRI mouse model pre-treatment with one of these oligopeptides, namely AQGV (EA-230), increased survival and tubular epithelial cell regeneration when treatment was given prior to IRI [13]. Since EA-230 showed good therapeutic effects in the IRI model we tested the hypothesis that EA-230 might also improve renal perfusion and attenuate IRI and ischemic allograft damage after allogenic kidney transplantation in mice.

## Materials and Methods

### Animals

The animal protection committee of the local authorities (Lower Saxony state department for food safety and animal welfare LAVES) approved all of our experiments (approval: 33.9-42502-04-09/1637). Inbred male C57Bl/6N (H2^b^) and female BALB/c (H2^d^) ten to twelve weeks old mice weighing between 20 g and 25 g were supplied by Charles River (Sulzfeld, Germany). Animals were cared for in accordance with the institution’s guidelines for experimental animals and with the guidelines of the American Physiological Society. Mice were housed under conventional conditions in individually ventilated cages (Techniplast Inc., Italy) with a 12h light/dark cycle, and had free access to food (Altromin 1324 standard mouse diet) and domestic quality drinking water. Animals were housed in groups of maximal 4 mice in a type II cage with a height of 13 cm in accordance with the Animal Unit’s Standard Operating Procedure and given environmental enrichment: Nesting material, chewing sticks, and mouse tunnels 10 cm long and 5.5 cm in diameter in each cage. After surgery mice were monitored daily for state of health and activity. Studies were terminated if mice showed obvious behavioral changes (e.g. reduced activity and food intake), high s-creatinine levels, or body weight reductions of >20% from baseline. Mice were sacrificed by being anesthetized with isoflurane and then perfused with cold PBS.

### Ischemia reperfusion injury to induce acute kidney injury (AKI)

Male C57Bl/6 mice were anesthetized with isoflurane and and were given butorphanol i.p. as needed during surgery. After median laparotomy the renal pedicle was bluntly dissected and a non-traumatic vascular clamp was applied to the renal pedicle for 35 min bilaterally. After removal of the clamps the abdomen was closed by suturing in two layers. Mice were returned to their cages and monitored until they were fully awake. Survival and renal function (serum (s)-creatinine and BUN) were studied in n = 16 mice and histology was performed in an additional n = 10 mice in each group two days after IRI. Treatment with EA-230 was started 24 hours after IRI with doses of 20, 30, 40 and 50mg/kg i.p. and continued for four consecutive days twice daily. To show that renal damage was similar in all groups prior to the first treatment, blood was drawn 24h after IRI and s-creatinine was measured.

### Kidney transplantation

Fully mismatched kidney transplantation was performed using C57Bl/6N (H2^b^) mice as kidney donors and BALB/c (H2^d^) mice as recipients. For isogenic transplantation C57Bl/6N mice served as donors and recipients. In previous studies we showed that male donor kidneys have higher s-creatinine elevations in the allogenic kidney transplant model than female donor organs shortly after ktx. The gender of the recipient did not influence s-creatinine elevation in this model. Therefore, to promote renal damage, all studies were performed with male donor kidneys and female recipients. Surgery was performed as described previously [14,15]. Briefly, animals were anesthetized with isoflurane and the left donor kidney was attached to a cuff of the aorta and the renal vein with a small vena cava cuff, and the ureter was removed en bloc. After left nephrectomy of the recipient, the vascular cuffs were anastomosed to the recipient abdominal aorta and vena cava, respectively, below the level of the native renal vessels. The ureter was directly anastomosed to the bladder. Cold ischemia time (CIT) of the grafts was prolonged to 60 min and warm ischemia time was 30 min, respectively. The right native kidney was removed through a flank incision four days after transplantation to have a life supporting kidney transplant model. As technical controls, isogenic transplantations were performed. For the kidney transplant experiments, recipients were treated one hour prior to surgery, a second time six hours after surgery and on the next three days, twice daily with either EA-230 (50mg/kg) i.p. or saline as vehicle (n = 10 ktx recipients in each group). Survival was monitored daily and kidney function weekly. Renal function parameters (s-creatinine and BUN) were measured using an Olympus AU 400 analyzer (Beckman Coulter Inc, USA).

### EA-230 compound and treatment strategy

EA-230 is a synthetic tetrapeptide Ala-Gln-Gly-Val (AQGV) which is a potential treatment for sepsis and inflammation. Its solution-based synthesis was performed by Diosynth BV (Oss, The Netherlands). EA-230 was diluted in saline prior to use.

In IRI experiments EA-230 was administered in doses of 20, 30, 40 or 50mg/kg i.p. twice daily over four consecutive days. The first treatment was given 24h after surgery once s-creatinine elevation due to AKI was already present in the IRI model. Physiological saline was used as vehicle. In studies of renal blood flow and glomerular filtration rate, EA-230 pre-treatment with 30mg/kg was given one hour prior to IRI and at 12 and 24 hours after IRI i.p.

In the kidney transplantation (ktx) experiments EA-230 (50mg/kg) treatment was given to recipients one hour pre-operatively, at six hours after surgery and subsequent treatments were given twice daily over four consecutive days.

### Renal blood flow and glomerular filtration rate measurements

Renal blood flow (RBF) and glomerular filtration rate (GFR) were evaluated 28h after IRI surgery in fully anesthetized mice by measuring of para-aminohippurate (PAH) and inulin clearance, respectively. EA-230 (30mg/kg) or saline treatment was given i.p. one hour prior to IRI and 12 and 24 hours after IRI. The solution containing inulin and PAH was infused into the tail vein after placing a catheter into the bladder. The infusion solution containing 15% inulin, 3.75% PAH and 1% bovine albumin (Sigma Chemical Co) in saline was infused at a rate of 5 μl/min per 100 g body weight. After a one hour equilibration period, urine was collected for 90 min and blood was drawn at the end of the infusion. Inulin and PAH concentrations in urine and plasma aliquots were measured by standard colorimetric methods.

### CTGF expression measured by Western blotting

To measure CTGF in kidney samples at day 28, tissue was snap frozen in liquid nitrogen and total protein was extracted as previously described. Relative expression of CTGF was calculated using GAPDH as the internal standard [16].

### Renal Histology

Formalin fixed and paraffin embedded kidneys were serially sectioned (3 μm) and stained with periodic acid Schiff (PAS) reagent. Acute tubular necrosis (ATN) consisted of a spectrum from loss of brush border as early lesion to overt cell necrosis with detachment from the basement membrane and accumulation of cell debris in the tubular lumen. Evaluation of acute tubular necrosis in the IRI tissue as well as in the kidney grafts was performed using a semi-quantitative grading system: 0 = no ATN, 1 = focal ATN with <10% of tubuli of the cortex affected, 2 = mild ATN with 10–25% of tubuli affected, 3 = moderate ATN with 25–50% of tubuli affected, 4 = severe ATN with >50% of the tubuli affected. Analysis was done by a nephropathologist without knowledge of the animal group identity. Ki-67 immunostaining (1:25, Dako, Germany) on paraffin sections was used to assess cellular proliferation. Positive nuclei were counted in ten different view fields of the cortex and the outer medulla per section (200x magnification) for comparison between groups. Inducible NOS (i-NOS; rabbit anti-mouse, 4mg/ml, 1:100, ABR) and endothelial NOS immunostaining (e-NOS; rabbit anti-mouse, 0.2mg/ml, 1:50, ABR) was performed on paraffin sections. Analysis of i-NOS and e-NOS was done by semi-quantitative counting of NOS positive glomeruli in 20 randomly chosen, non-overlapping fields of the cortex in 200x magnification. Semi-quantitive score: 0 = no expression, 1 = weak (<10% of the glomeruli), 2 = moderate (10–30%), 3 = high (30–50%) expression.

### Statistical analysis

Data are shown as mean ± standard error (SEM). Statistical significance (*p<0.05, **p<0.01, ***p<0.005) was calculated by ANOVA and post hoc Scheffe Test for independent groups. For survival analysis log rank test was used. Statistical analysis was performed with Graphpad prism version 6.0.

## Results

### EA-230 treatment improved survival and renal function after IRI

In previous studies a beneficial effect of several beta hCG derived oligopeptides in IRI was shown when treatment was given prior to IRI [13]. Here, we tested the tetrapeptide EA-230 starting 24h after IRI when the initial damage, indicated by similar increases in s-creatinine in all groups, were already present. EA-230 was given to n = 16 mice at 20, 30, 40, or 50mg/kg i.p. twice daily for four consecutive days.

**Figure 1:**
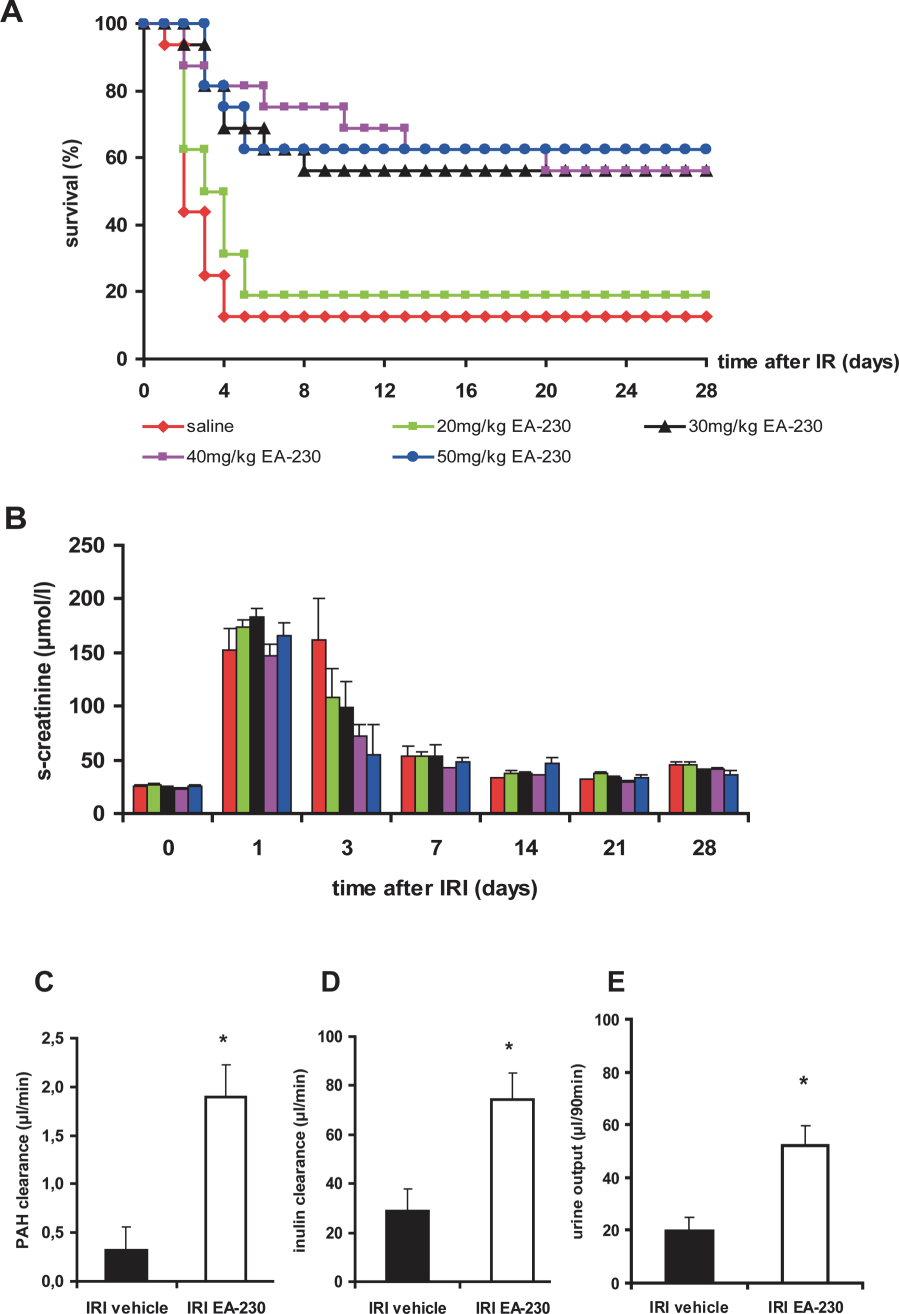
IRI was performed and 24h after surgery EA-230 or vehicle treatment was given twice daily i.p. for four consecutive days. The majority of vehicle treated mice died within four days after IRI (A). EA-230 treated mice receiving doses between 30 and 50mg/kg survived significantly better than vehicle treated mice (***p<0.005). Renal function measured by s-creatinine (B) showed the initial increase and normalized over time in the surviving mice of all groups. Renal blood flow (C, PAH clearance), glomerular filtration rate (D, inulin clearance) and urine output (E) were significantly higher in EA-230 treated mice (*p<0.05).

Post-damage treatment with EA-230 improved survival markedly (Fig. 1A). With EA-230 doses between 30–50 mg/kg survival reached 56–62% (p<0.005 compared to vehicle); in the vehicle treated group only 12.5% of mice survived for four weeks. The low dose treatment with 20mg/kg did not have a beneficial effect. In all groups s-creatinine elevation (Fig. 1B) was six fold increased 24h after IRI (prior to treatment initiation). At day three s-creatinine levels in the EA-230 treated groups appeared to be lower than in the vehicle treated group but the difference did not reach significance. In all surviving mice, s-creatinine elevation declined almost to baseline levels within the first week after IRI.

### EA-230 improved microcirculation and attenuated acute tubular damage

**Figure 2:**
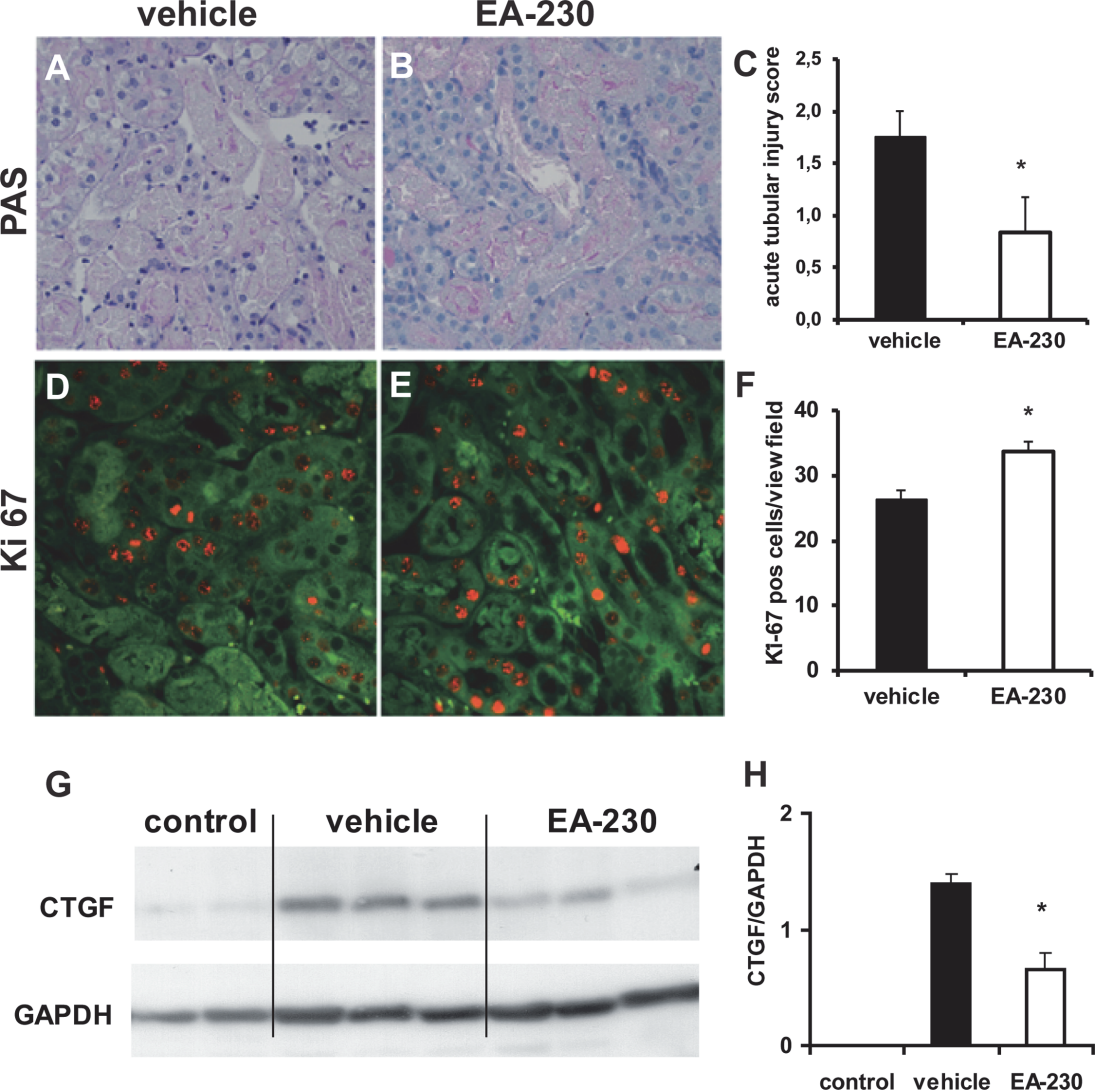
Acute tubular necrosis (A-C) two days after IRI affected ∼25% of tubuli in the vehicle treated mice and was less in the EA-230 treated mice (A-C, magnification 200 fold). The number of Ki-67 positive tubular epithelial cells as marker of proliferation (red staining) was significantly higher in EA-230 treated kidneys two days after IRI (D-F, magnification 200fold, the autofluorescent kidney tissue appears green).

To determine whether better survival was associated with improved renal blood flow (RBF) and renal function, PAH and inulin clearance measurements were performed. EA-230 treatment significantly increased RBF (Fig. 1C, *p<0.05). In addition, glomerular filtration rate (GFR) as measured by inulin clearance (Fig. 1D, *p<0.05) and urine output during the 90min collection period (Fig. 1E, *p<0.05) were significantly higher in EA-230 treated mice than in vehicle treated controls after IRI. Tissue damage two days after IRI was minimal in the EA-230 treated mice which had only mild acute tubular necrosis (ATN) with fewer than 10% of tubuli affected. In contrast, vehicle treated animals had moderate to severe ATN (∼25% tubular damage two days after IRI; Fig. 2 A-C, *p<0.05).

In renal tissue of surviving mice at four weeks after IRI the fibrosis marker CTGF was clearly up-regulated in vehicle treated mice. EA-230 treatement attenuated CTGF up-regualation (G, H). Representative Western blots of each groups are shown (experiment was performed twice, reletive expression compared to GAPDH was calculated).

### EA-230 enhanced tubular epithelial cell regeneration

Immunostaining Ki-67 revealed that at two days after IRI significantly more tubular epithelial cells were proliferating in the kidneys of EA-230- than in the kidneys of vehicle-treated mice (Fig. 2 D-F, *p<0.05).

### EA-230 attenuated TGF-beta activation

TGF-beta activation is observed in IRI and contributes to renal fibrosis and chronic kidney damage (CKD). To investigate the effect of EA-230 on TGF-beta activation, its target, CTGF, was measured by western-blotting four weeks after IRI. EA-230 treatment attenuated CTGF up-regulation in the kidney significantly (Fig. 2 G, H, *p<0.05).

### EA-230 prevented ischemic allograft damage after kidney transplantation

**Figure 3:**
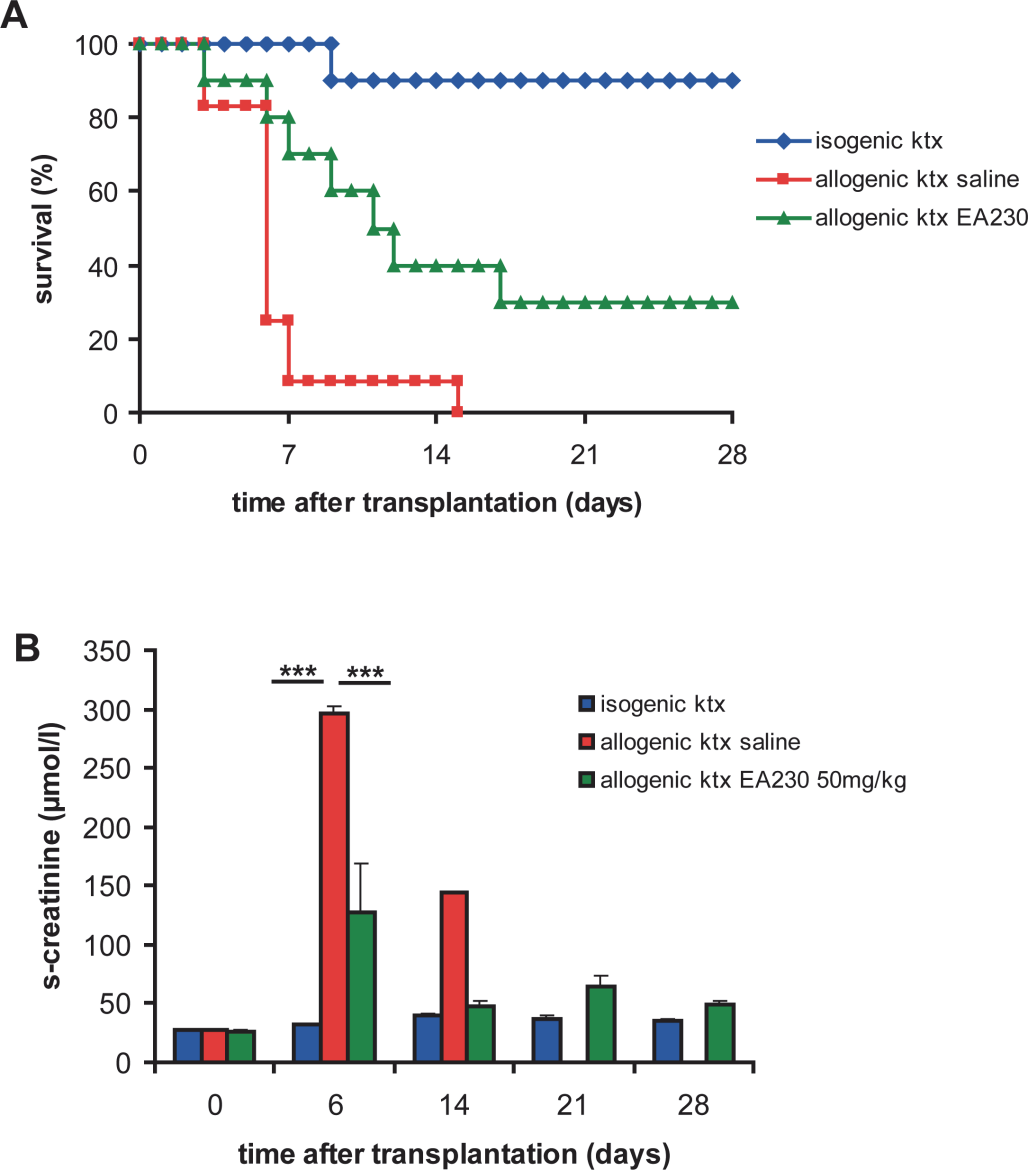
Isogenic transplantation (blue) revealed 90% survival for four weeks and normal renal function (B). After allogenic kidney transplantation 90% of the vehicle treated mice died within seven days after nephrectomy of the contralateral native kidney of the recipients at day 4. In contrast 70% of EA-230 treated mice were still alive one week after ktx. 30% of the recipients treated with EA-230 achieved four week survival whereas all vehicle treated recipients died within the first two weeks. Renal function (B) deteriorated at day six in both allogenic ktx groups but was significantly better in EA-230 (green columns, ***p<0.005) than in vehicle treated allograft recipients (red columns).

**Figure 4:**
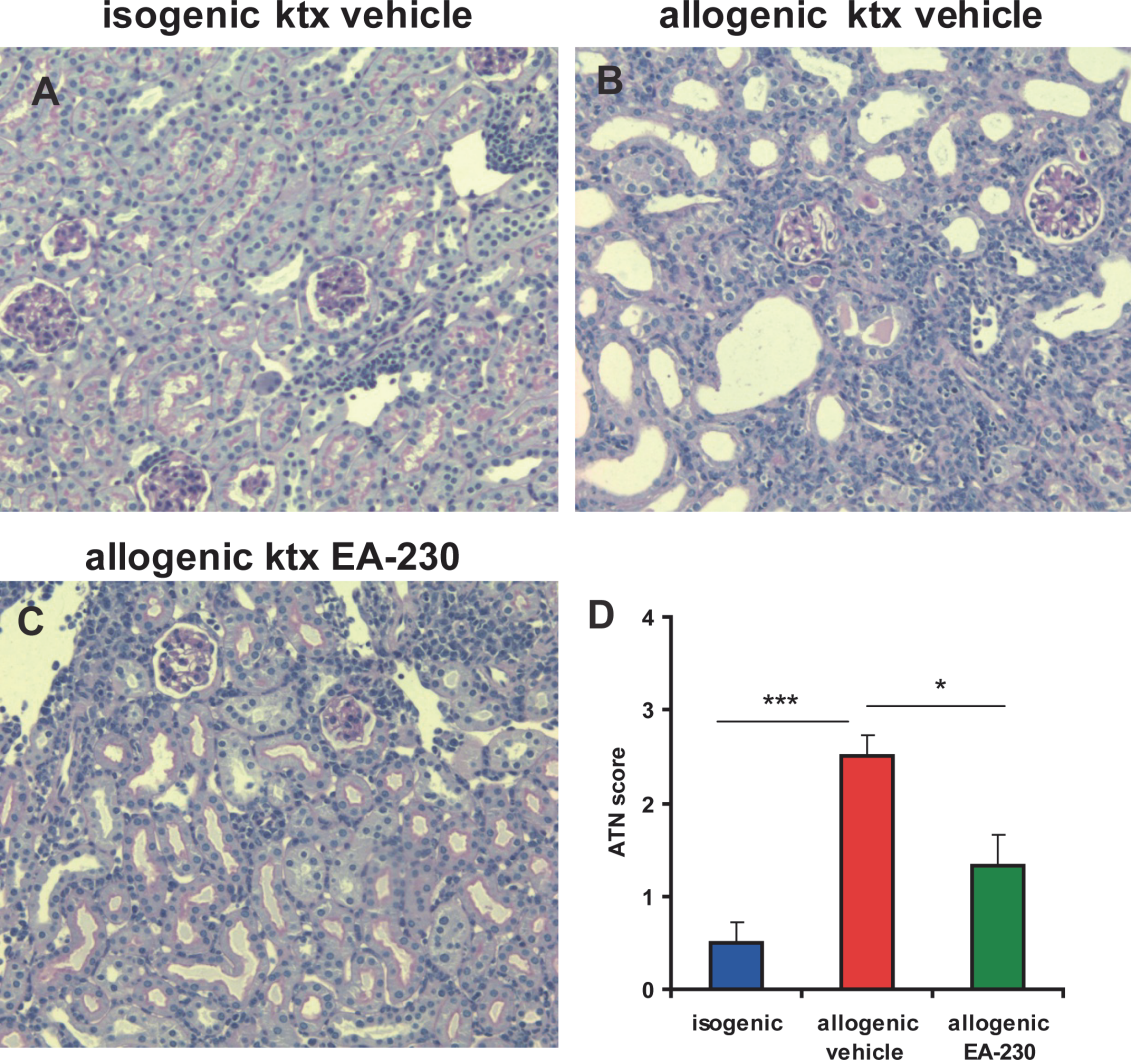
PAS stained isografts (A) and allografts with vehicle treatment (B) or EA-230 50mg/kg (C) treatment six days after transplantation. Isografts had no signs of acute rejection or acute tubular necrosis. Vehicle treated allografts showed severe ATN with 50% of the tubuli affected and Banff 1A acute rejection with interstial infiltrates, tubulitis and gomerulitis (B). EA-230 treated allografts (C) showed mild ATN with ∼10% of tubuli affected and borderline rejection (magnification 200 fold, D semiquantitative score for ATN).

To determine whether EA-230 can also attenuate ischemic allograft damage we transplanted C57Bl/6 donor kidneys exposed to prolonged cold ischemia times (60min) into BALB/c recipients. EA-230 treatment (50mg/kg i.p.) of the recipient was started two hours prior to transplantation and continued for four days twice daily. A life-supporting allogenic kidney transplant model was used in which the remaining native kidney of the recipient was removed after four days after ktx in order to study survival and renal function of the allograft (Fig. 3). Ninety percent of the vehicle treated recipients died within seven days of allogenic ktx. In contrast, 40% of EA-230 treated allograft recipients survived the first week without additional immunosuppressive therapy. In the isogenic control group survival was 90% over the whole observation period of four weeks (Fig. 3A). Due to severe ischemic injury and subsequent inflammation, vehicle treated allograft recipients showed severe loss of renal function with a significant s-creatinine elevation six days after transplantation (297 ± 5 μmol/l, ***p<0.005 vs baseline). This increase in s-creatinine was markedly attenuated in EA-230 treated allograft recipients (106 ± 7 μmol/l; ***p<0.005 vs. vehicle; Fig. 3B). In further experiments 10 additional allografts in each group were analysed for acute tubular necrosis (ATN) six days after transplantation. In vehicle treated allograft tissue moderate to severe ATN affected ∼50% of the tubuli. EA-230 treatment revealed better outcome in the allografts with only focal ATN affecting <10% of the tubuli (Fig. 4). The vehicle treated allografts showed Banff 1A rejection, in the EA-230 treated group borderline rejection was detectable.

## Discussion

In this study we investigated a novel therapeutic approach to improve outcome after IRI and ischemic allograft damage. We tested a tetrapeptide, EA-230, which has been shown previously to attenuate multi-organ failure in sepsis [11] and to improve survival in IRI- induced acute kidney injury in mice when treatment was given prior to renal pedicle clamping [13]. In the current study, we showed that higher and repeated dosing of the EA-230 significantly improves survival even when treatment was delayed for 24h after IRI when the acute kidney injury with s-creatinine elevation had already occurred. We speculated that the beneficial effects of EA-230 were related to amelioration of renal perfusion and enhanced tubular epithelial cell proliferation and regeneration.

Indeed, we could show that in the IRI model EA-230 improved renal blood flow significantly and that GFR was enhanced markedly. In previous magnetic resonance (MR) imaging studies we could show by arterial spin labeling that renal cortical perfusion decreased dramatically within 24h of IRI and continued to decrease to only 60% of normal seven days after IRI in C57Bl/6N mice. Renal perfusion impairment correlated significantly with kidney volume loss and renal tubulo-interstitial fibrosis within four weeks [9]. Furthermore, we could show that along with disturbances of renal microcirculation, capillary leakage and edema formation increased until day seven in this model [17] and both correlated with the duration of ischemia time. After prolonged ischemia (IRI for 45min) the impairment of renal blood flow and the extent of edema formation were quite pronounced and predicted the later progression to chronic kidney disease (CKD)[10]. Based on our previous studies we think that improved renal perfusion is an important mode of action of EA-230. Furthermore, we could show that delaying treatment with until 24h after IRI EA-230 still enhanced tubular epithelial cell regeneration markedly. This is consistent with a previous study showing enhanced tubular epithelial cell regeneration one day after IRI when EA-230 treatment was given prior to the injury [13]. We could also show that EA-230 treatment attenuated TGF-beta induced up-regulation of the fibrosis marker CTGF four weeks after IRI. Recently, it has been shown in a transgenic mouse model with inducible constitutive activation of the TGF beta receptor 1 in tubular epithelial cells that local TGF-beta activation alone caused AKI and tubular inflammation resulting in enhanced renal fibrosis [18]. In renal disease models such as glomerulonephritis and hypertensive renal damage it has also been shown that chronic tissue hypoxia due to impaired microcirculation and peritubular capillary loss precedes progression of tubulo-interstial fibrosis to chronic kidney disease [19–21]. In human transplant studies Snoeijs and coworker showed that in deceased cadaveric donor (DCD) transplantation in the early reperfusion period microvascular perfusion was 42% lower than in living donor kidneys. DCD grafts had smaller blood vessel diameters and reduced capillary blood flow [3]. Kidney transplant biopsy studies revealed that low numbers of peritubular caplillaries correlated significantly with the development of interstitial fibrosis and graft dysfunction [22]. Since EA-230 improved renal blood flow and attenuated renal fibrosis in the IRI model markedly we wondered whether EA-230 could also attenuate ischemic allograft injury. We showed that a four day treatment with EA-230 improved renal function and survival after allogenic kidney transplantation significantly. In addition, acute tubular necrosis was also significantly attenuated compared to that seen in vehicle treated mice. We hypothezise that the beneficial effects of EA-230 on renal perfusion might be responsible for markedly better outcome in the model of ischemia induced kidney allograft damage [15]. In previous studies, we could identify a similar protective mechansim of PKC epsilon deficiency which improved renal perfusion and attenuated AKI and kidney allograft rejection [23]. The unique microvaculature of the kidney, associated with a high oxygen demand mainly in proximal tubuli of the outer medulla makes the kidney highly sensitive to hypoxia. Microcirculatory alterations cause local hypoxia and activation of pro-inflammatory pathways [24]. Enothelial nitric oxide synthetase (e-NOS) derived nitric oxide is essential for sustaining renal oxygen supply and acts in a paracrine fashion by direct vasodilatory effects, inhibiting platelet aggregation and leukocyte acitvation [25]. On the other hand, ischemia induced activation of inducible (i-) NOS participates in vascular dysfunction. Several strategies of inhibition of i-NOS as well as up-regulation of e-NOS have been experimentally adressed and shown to improve renal perfusion [26]. In addition, blockers of beta-1 and beta-3 adrenergic receptors expressed on renal vascular and glomerular endothelial cells cause vasodilatation via NO dependent mechanisms [27,28]. In our treatment protocol with delayed initiation of EA-230 treatment at 24h after IRI we could not show any differences in renal e-NOS or i-NOS expression during the first three days (S1 Fig.). This might be due to the fact that NO signalling is up-regulated early after IRI and was already acitvated 24h after IRI. In preventive treatment strategies with treatment prior to injury the NO release might also explain renal vasodilatation and better renal perfusion, however, the exact mechanism by which EA-230 improves renal perfusion when given post-damage and whether this may be related to NO release is not known. Further, evidence of EA-230’s mode of action has been investigated in previous studies showing that EA-230 amongst other oligopeptides prevented multiorgan failure in sepsis [11]. In these studies, the beta-hCG derived oligopeptides reduced neutrophile influx, release of pro-inflammatory cytokines (i.e. TNF-alpha, IL-6, INF-gαmma) and up-regulation of adhesion molecules. This early anti-inflammatory mode of action is relevant when EA-230 treatment can be initiated prior to injury (e.g. prior to major cardiac surgeries or transplantation). Collectively, the results of the current study and the previously described beneficial effects in experimental sepsis models encourage the use of EA-230 in transplantation with the aim to improve microcirculation of the renal allograft and to diminish ischemic allograft damage. In combination with standard immunosuppressive therapy, short term EA-230 treatments might reduce the rate of delayed graft function (DGF) and the need for dialysis, and might also reduce tubulo-interstitial fibrosis and tubular atrophy in patients who receive kidney grafts with prolonged cold ischemia times. Furthermore, in major cardiac surgeries, or organ transplantations with increased risk of acute kidney injury, EA-230 treatment might also improve renal outcome and could be given peri-operatively in addition to the current therapeutic protocol.

## Supporting Information

S1 Fig Endothelial NOS (e-NOS, A: IRI vehicle, B: IRI EA-230, C: semiquantitative scoring) and inducible NOS (i-NOS, D: IRI vehicle, E: IRI EA-230, F: semiquantitative scoring) expression was up-regulated in the glomeruli of vehicle as well as of EA-230 treated mice after IRI and remained high over the first three days. Representative images of day 2 are shown. No difference between groups was observed (bar represents 10μm, magnification 400 fold, scoring 0- no expression, 1- mild, 2-moderate, 3-high expression).
(EPS)
